# Supplementary material for: Significant Factors for Modelling Survival of Escherichia coli in Lake Sediments
Source: Microorganisms. 2024 Jun 13;12(6):1192. doi: 10.3390/microorganisms12061192 (PMC11206117; doi:10.3390/microorganisms12061192)
Supplement: Supplementary file 1 [file microorganisms-12-01192-s001.zip › microorganisms-3039578-supplementary.pdf]

Significant Factors for Modelling Survival of *Escherichia coli* in Lake Sediments

Ichiro Yoneda [1], Masateru Nishiyama [2] and Toru Watanabe [2,*]

[1] Department of Regional Environment Creation, United Graduate School of Agricultural Sciences, Iwate University, 18-8 Ueda 3-Chome, Morioka 020-8850, Japan.

[2] Department of Food, Life and Environmental Sciences, Faculty of Agriculture, Yamagata University, 1-23 Wakaba-Machi, Tsuruoka 997-8555, Japan.

* Corresponding author: Toru Watanab; to-ru@tds1.tr.yamagata-u.ac.jp

Table S1 Physico-chemical properties of sediments used in this study. Sediment component was determined by hydrometer method [51]. Moisture and organic matter contents were determined by loss on drying and loss of ignition method, respectively [52]. Water-extractable DOC content was measured using TOC-L (SHIMADZU, Kyoto, Japan) after air-dried sediment sample was mixed with Mili-Q water and passed through a polyethersulfone membrane filter [35]. *E. coli* and total coliform were measured using the dilution plate method [37] with Chromocult Coliform Agar (Merck, Darmstadt, Germany).

Sample name	Treatment	Texture type	Sediment component (%)			Moisture content (%)	Organic matter (%)	Water-extractable DOC (mg/g-wet)	*E. coli* (CFU/g-wet)	Total coliform (CFU/g-wet)
			Clay	Silt	Sand					
Lake A	Untreated	Sandy loam	9.4	38.3	52.4	75.0	13.1	0.045	8.9	5.9×10^2
Lake B	Untreated	Loam	12.8	40.4	46.8	77.9	15.7	0.029	<LOD	1.4×10^3
Lake C	Untreated	Sandy loam	7.5	29.6	63.0	76.1	8.7	0.019	15.5	4.6×10^3
Lake A	Autoclaved	Loam	12.6	38.2	49.1	75.3	13.2	0.093	<LOD	<LOD
Lake B	Autoclaved	Loam	18.2	43.5	38.3	77.9	17.3	0.077	<LOD	<LOD
Lake C	Autoclaved	Sandy loam	7.2	32.5	60.3	74.8	13.1	0.116	<LOD	<LOD

LOD indicates low of detection.

Table S2 Compositions of water-extractable TDS separated from lake sediment collected on 1 November 2023. Anions (F^-, Cl^-, Br^-, NO_2^-, NO_3^-, PO_4^{3-}, SO_4^{2-}) and cations (Li^+, Na^+, NH_4^+, K^+, Ca_2^+, Mg_2^+) were measured using HIC-ESP/NS (SHIMADZU, Kyoto, Japan).

Added DOC level (mg)		0.004	0.01
DOC	mg/L	40.0	104.2
DTN	mg/L	3.3	8.7
F^-	mg/L	0.11	0.29
Cl^-	mg/L	27.8	38.4
Br^-	mg/L	0.0002	0.0003
NO_2^-	mg/L	0.06	0.15
NO_3^-	mg/L	0.11	0.28
PO_4^{3-}	mg/L	0.002	0.02
SO_4^{2-}	mg/L	39.1	110.3
Li^+	mg/L	N.D.	N.D.
Na^+	mg/L	24.1	40.9
NH_4^+	mg/L	0.05	0.16
K^+	mg/L	2.37	5.67
Ca^{2+}	mg/L	2.55	6.55
Mg^{2+}	mg/L	2.39	6.10

N.D. indicates not detected.

Table S3 Regression coefficients for each factor determined by multiple linear regression analysis using the *E. coli* population change rate and each factor as dependent and independent variables, respectively. Before the multiple linear regression analysis, the values of each factor were standardized. R(version4.2.2) was used for this analysis.

Parameter	Coefficient
Temperature	-0.14
pH	-0.44*
Water-extractableTDS	-0.13
Coexistingmicrobes	0.02
Samplingsites	0.02

*p<0.05.

References

35. NandaKafle, G.; Christie, A.A.; Vilain, S.; Brözel, V.S. Growth and extended survival of Escherichia coli O157: H7 in soil organic matter. Front. Microbiol. 2018, 9, 762.

37. Prayoga, W.; Nishiyama, M.; Praise, S.; Pham, D.V.; Van Duong, H.; Pham, L.K.; Dang, L.T.T.; Watanabe, T. Tracking fecal bacterial dispersion from municipal wastewater to peri-urban farms during monsoon rains in hue city, vietnam. Int. J. Environ. Res. Public Health 2021, 18, 9580.

51. Barman, U.; Choudhury, R.D. Soil texture classification using multi class support vector machine. Inf. Process. Agric. 2020, 7, 318–332.

52. Zhao, Q.; Li, R.; Ji, M.; Ren, Z.J. Organic content influences sediment microbial fuel cell performance and community structure. Bioresour. Technol. 2016, 220, 549–556.
